# The association between incidence and mortality of brain cancer and human development index (HDI): an ecological study

**DOI:** 10.1186/s12889-020-09838-4

**Published:** 2020-11-12

**Authors:** Zaher Khazaei, Elham Goodarzi, Vahidreza Borhaninejad, Farhad Iranmanesh, Hosein Mirshekarpour, Batool Mirzaei, Hasan Naemi, Sayeed Maryam Bechashk, Isan Darvishi, Roghayeh Ershad Sarabi, Ahmad Naghibzadeh-Tahami

**Affiliations:** 1 Department of Public Health, School of Medicine, Dezful University of Medical Sciences, Dezful, Iran; 2 Social Determinants of Health Research Center, Lorestan University of Medical Sciences, Khorramabad, Iran; 3 Social Determinants of Health Research Center, Institute for Futures Studies in Health, Kerman University of Medical Sciences, Kerman, Iran; 4 Professor of Neurology, Stroke Fellowship, Neuroscience Research Center, Institute of Neuropharmacology, Kerman University of Medical Sciences, Kerman, Iran; 5 Clinical Research Unit, Shafa Hospital, Kerman University of Medical Sciences, Kerman, Iran; 6 Endocrinology and Metabolism Research Center, Institute of Basic and Clinical Physiology Sciences, Kerman University of Medical Science, Kerman, Iran; 7 Iranian Research Center on Healthy Aging, Sabzevar University of Medical Sciences, Sabzevar, Iran; 8 Epidemiology, Social Determinants of Health Research Center, Research institute for Health Development, Student Research Committee, Kurdistan University of Medical Sciences, Sanandaj, Iran; 9 Department Of Operating Room, Instructor Of Operating Room ,Shahrekord University Of Medical Sciences, Shahrekord, Iran; 10 Medical Informatics Research Center, Institute for Futures Studies in Health, Kerman University of Medical Sciences, Kerman, Iran; 11 Modeling in Health Research Center, Institute for Futures Studies in Health, Kerman University of Medical Sciences, Kerman, Iran

**Keywords:** Incidence, Mortality, Brain Cancer, Human development index, World

## Abstract

**Background:**

Brain cancer is a rare and deadly malignancy with a low survival rate. The present study aims to evaluate the epidemiology of brain cancer and its relationship with the human development index (HDI) worldwide.

**Methods:**

This is an ecological study. The data on cancer incidence and cancer mortality was extracted from the World Bank for Cancer in 2018 (GLOBOCAN 2018). The incidence, mortality rate, and brain cancer distribution maps were drawn for different countries. We used correlation and regression tests to examine the association of incidence and mortality rates of brain cancer with HDI. The statistical analysis was carried out by Stata-14 and a significance level of 0.05 was considered.

**Results:**

According to the results of Global Cancer Registry in 2018, there were 18,078,957 registered cases of cancer in both sexes, of which 29,681 were related to brain cancer. The highest incidence (102,260 cases, 34.4%) and mortality (77,815 cases, 32.3%) belonged to very high HDI regions. Results showed that incidence (*r* = 0.690, *P* < 0.0001) and mortality rates (*r* = 0.629, *P* < 0.001) of brain cancer are significantly correlated with HDI. We also observed a positive correlation between brain cancer incidence and Gross National Income (GNI) (*r* = 0.346, *P* < 0.001), Mean Years of Schooling (MYS) (*r* = 0.64, *P* < 0.001), TABLE (LEB) (*r* = 0.66, *P* < 0.001) and Expected Years of Schooling (EYS) (*r* = 0.667, *P* < 0.001). Results also revealed that mortality rate was significantly correlated with GNI (*r* = 0.28, *P* < 0.01), MYS (*r* = 0.591, *P* < 0.01), LEB (*r* = 0.624, *P* < 0.01), and EYS (*r* = 0.605, *P* < 0.01).

**Conclusion:**

The results of the study showed that the incidence and mortality of brain cancer in countries with higher HDI levels is higher than countries with lower HDI levels, so attention to risk factors and action to reduce it in countries with higher HDI levels in controlling this cancer in this Countries are effective.

## Background

Today, non-communicable diseases (NCDs) are a leading cause of death worldwide [1]. According to WHO estimates, cancer was the first or second leading cause of mortality in people before under 70 years of age in most countries worldwide in 2015 [2]. Population growth, population aging and economic development are the main reasons for rising prevalence of cancer worldwide [3, 4]. Cancers are the leading cause of death in some developed countries and the second major cause of death after cardiovascular disease in developing countries [5]. Brain cancer is a major cancer whose prevalence has been on rise in recent decades [6]. Malignant brain tumors are relatively rare, accounting for only 1–2% of all types of cancer in adults [7]. According to GLOBOCAN 2012, brain cancer is one of rare types of cancers around the world. Considering the low survival of patients with brain cancer and its impact on patients’ quality of life, economic costs, and mortality rate, it demands special attention [6, 8].

The main factors in the development of brain cancer are exposure to ionizing radiation, pesticides, and cyclic aromatic hydrocarbons [9]. Environmental and socioeconomic factors influence mortality induced by brain cancer [10–14]. The discrepancy in brain cancer incidence around the world is due to factors such as causative factors, improved diagnostic methods and enhanced medical care in different countries [15].

There is tremendous variation in the detection rate of brain cancer’s new cases worldwide [16]. The global demographic and epidemiological trends indicate a dramatic increase in cancers over the coming decades, especially in low- and middle-income countries [17]. The incidence of brain tumors is higher in the Western countries than in the Eastern countries. It is also higher in developed countries than in developing countries. The highest incidence of brain tumor has been observed in Australia, North America and Northern Europe and the lowest incidence in Africa [18].

According to the latest report from the National Vital Statistics System for the period of 1975 to 2016, the mortality rate has been higher among men and old people [19]. The incidence of brain cancer in developed countries is higher than in developing countries [18]. However, the highest rate of age-specific mortality of brain cancer can be found in developing countries [20]. The burden of brain cancer is also higher in areas with high HDI, according to a 1990 United Nations’ estimate of the Human Development Index (HDI). As a summary of human development system, HDI measures the basic dimensions of human development including a long and healthy life, education, and decent standards of living [21].

Given the global pattern of brain cancer and its timing in recent years, it is essential to raise public awareness about this cancer with the goal of planning and management of financial and human resources to prevent this cancer. Despite the growing burden of cancers in developing countries, they account only for 5% of global spending on cancers [22]. Each country is required to assess whether socioeconomic status has a bearing on cancer risk factors by comparing its data with that of other countries.

The goal of this study is to examine the impact of socioeconomic development (in terms of HDI) on global trends in the incidence and mortality of brain cancer worldwide based on data extracted from World Bank in 2018.

## Methods

Given the limited quality and coverage of existing cancer data in the world, especially in low-income or middle-income countries, it is necessary to be cautious in interpreting these data. The International Agency for Research on Cancer (IARC) approach is not just to evaluate, compile and use data from other institutions, but the intention of the centre is to work with country centres to improve the quality of native data, data coverage and analytical capacities. The urgent need for investing in the coverage of cancer-based population data in low-.and middle-income countries was to launch the Global Initiative for the Cancer Registry Development (GICR) in cooperation with the IARC. The GICR aims to provide information on cancer control, which can be regularly promoted through coverage, quality and use of population-based cancer data. A summary of the steps used to calculate the incidence of cancer, its mortality and its prevalence are presented below. The calculation methods vary from country to country, and the quality of the national computing data depends on the coverage, accuracy, time of the outbreak and the deaths at each country [5, 23].

### Incidence

In this ecological study, age and gender-related incidence rates in each country were calculated using the following steps: 1. the nationally monitored incidence rate reported by 45 countries until 2018; 2. The most recently observed national or regional incidence rates for the population of 50 countries in 2018; 3. The rates calculated from modeling national mortality data as well as mortality-to-incidence ratio obtained from the cancer registry of countries (14 countries); 4. The rates obtained from the estimates of national mortality using mortality-to-incidence ratios derived from cancer registries of neighboring countries (*n* = 37); 5. National incidence rates of all types of cancer with respect to gender and age obtained from calculating the average incidence rates in neighboring countries. The obtained values are then partitioned to achieve the national incidence rate for each specific site using relative cancer incidence data (7 countries), and 6. The rates calculated as the average rates of incidence in selected neighboring countries.

### Mortality

The age and gender-related cancer mortality rates in each country were calculated using the following steps: 1. Supervised national mortality rates reported in 81 countries) by 2018; 2. The most recent national mortality rates observed in 2018 used for the population of 20 countries; 3. Using relational modeling, mortality-to-incidence rates and mortality rates collected from cancer registry data in neighboring countries (*n* = 81); 4. The mortality rates calculated as the averages of mortality rate n selected neighboring countries (*n* = 3, [24, 25]).

### HDI

HDI comprises three dimensions of education, life expectancy and decent standards of living. The regions and countries with significant progress in all components of HDI have a faster and higher growth than countries with low or moderate HDI. According to this index, the world is unequal since the national averages do not reflect experiences of different individuals. There are huge inequalities in the North and South, with the intra- and inter-country inequality in income riding [26–28].

## Results

**Fig. 1:**
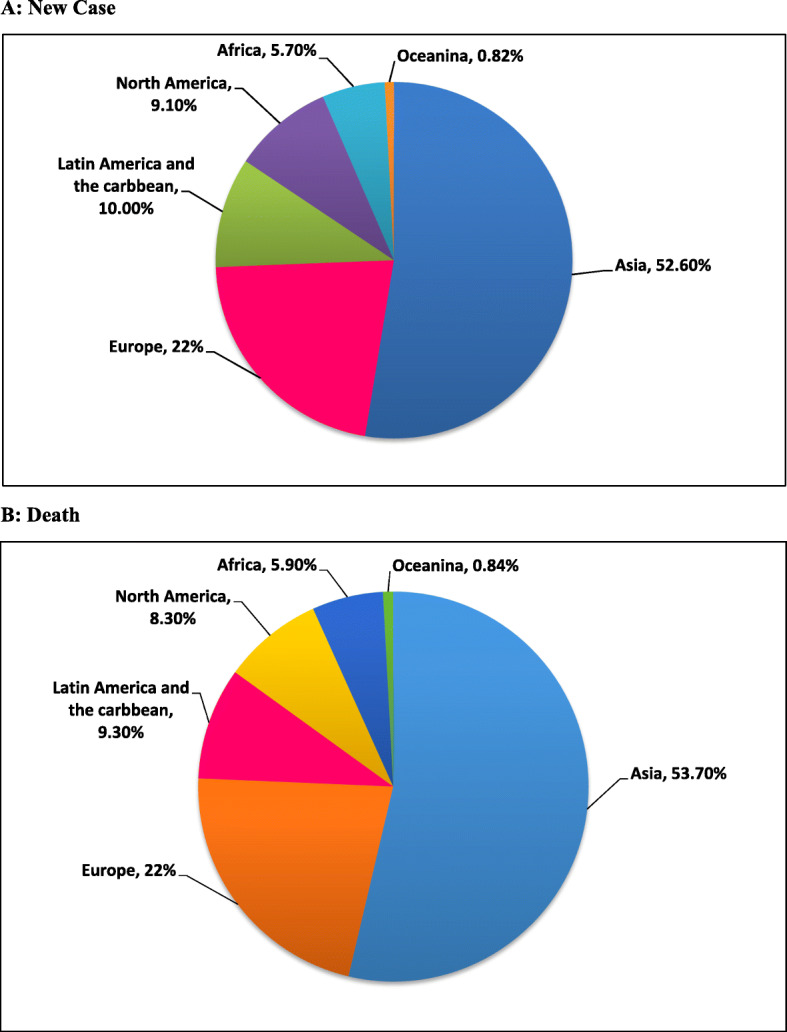
Pie charts present the distribution of brain cancer’s cases and deaths by continent in 2018 for both sexes and all ages. [Source: GLOBOCAN 2018]

According to the results of 2018 Cancer Registry, there are 18,078,957 registered cases of cancers in both sexes, of which 296,851 are related to brain cancers. The highest incidence of brain cancer was reported in Asia (156,217 cases, 52.6%) and the lowest incidence in Oceania (2438 cases, 0.82%). There were 9,555,027 cases of deaths attributable to global cancers in 2018, of which 241,037 (2.71%) belonged to brain cancers. The highest mortality rate was observed in Asia with 129,483 cases (57.3%) and the lowest mortality in Oceania with 2017 cases (0.84%) (Fig. 1, [29, 30]).

**Fig. 2:**
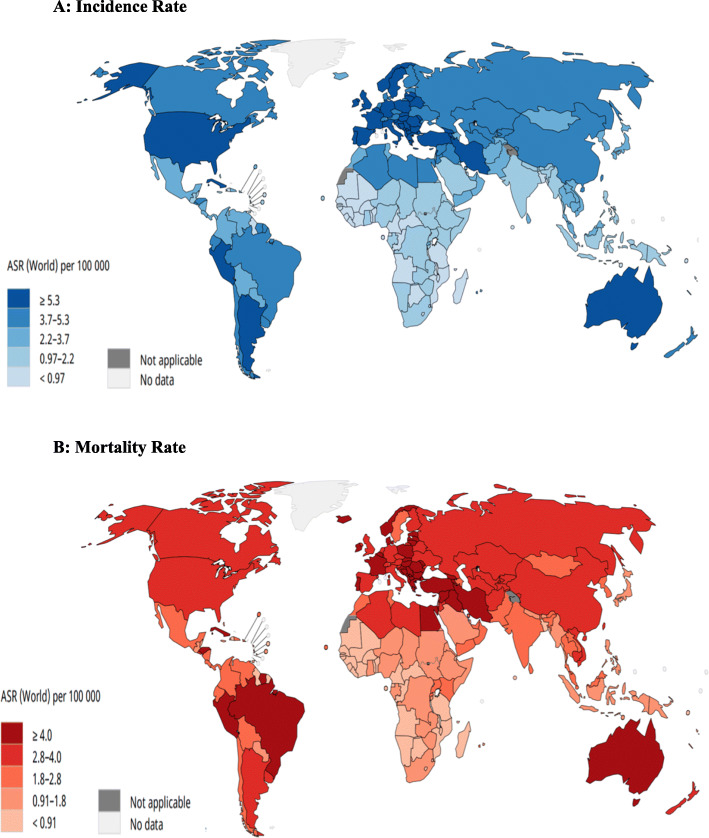
The map presenting **a** incidence and **b** mortality rates of brain cancer in both sexes and for all ages in in 2018. [Source: GLOBOCAN 2018]

According to the results, the highest incidence of brain cancer was observed in Latvia (10.1 per 100,000), the former Yugoslav Republic of Macedonia (9.2 per 100,000) and Lithuania (8 per 100,000), and the highest mortality rates in the former Yugoslav Republic of Macedonia (6.9 per 100,000), Armenia (6.1 per 100,000) and Albania (5.8 per 100,000), respectively (Fig. 2, [29, 30]).

The analysis of variance revealed that the highest mean incidence (5.08 per 100,000) was related to very high HDI and the lowest (0.9 per 100,000) to low HDI regions and this difference was statistically significant (*P* < 0.0001). The lowest mortality rate (0.9 per 100,000) was related to low HDI and the highest mortality rate (3.6 per 100,000) to very high HDI regions. This difference was also statistically significant (*P* < 0.0001) (Table 1).

**Table 1:** Pearson correlation between HDI components and the dependent variable

HDI	Incidence Rate		Mortality Rate	
	CR	ASR	CR	ASR
Very high human development	7.6	5.08	6.06	3.6
High human development	4.4	3.7	3.6	2.9
Medium human development	2.04	2.2	1.7	1.9
Low human development	0.7	0.9	0.6	0.9
*P*-value(F-test)	*P* < 0.0001	*P* < 0.0001	*P* < 0.0001	*P* < 0.0001

*Abbreviations*: *CR* Crude Rate, *ASR* Age-Standardized Rates per 100,000

**Fig. 3:**
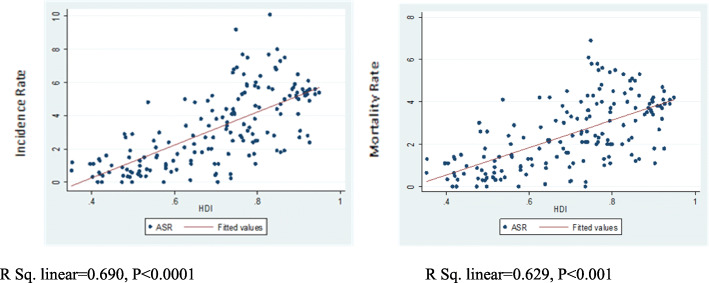
Correlation of HDI with incidence and mortality rates of brain cancer worldwide in 2018

The results suggested that incidence (*r* = 0.690, *P* < 0.0001) and mortality (*r* = 0.629, *P* < 0.001) of brain cancer were significantly correlated with HDI (Fig. 3).

Moreover, as shown by the results, there was a positive correlation between the incidence of brain cancer and (*r* = 0.346, *P* < 0.001), MYS (*r* = 0.64, *P* < 0.001), LEB (*r* = 0.66, *P* < 0.001) and EYS (*r* = 0.667). Results also manifested a significant positive correlation between mortality rate and GNI (*r* = 0.28, *P* < 0.01), MYS (*r* = 0.591, *P* < 0.01), LEB (*r* = 0.624, *P* < 0.01) and EYS (*r* = 0.605, *P* < 0.01) (Table 2).

**Table 2:** Relationship between incidence and mortality rate of brain cancer and HDI

HDI Component	ASIR^a^		ASMR*	
	r	*P*-value	r	*P*-value
Gross national income per 1000 capita	0.346	*P* < 0.0001	0.28	*P* < 0.0001
Mean years of schooling	0.64	*P* < 0.0001	0.591	*P* < 0.0001
Life expectancy at birth	0.66	*P* < 0.0001	0.624	*P* < 0.0001
Expected years of schooling	0.667	*P* < 0.0001	0.605	*P* < 0.0001

^a^ Dependent variables: ASIR and ASMR

## Discussion

The findings of this study demonstrated that the highest incidence of brain cancer in the World belonged to Latvia in continental Europe (10.1 per 100,000) and the highest mortality rate was related to the Former Yugoslav Republic of Macedonia (6.9 per 100,000) and Armenia, respectively. (6.1 per 100,000). In general, the highest incidence of brain cancer was reported in Asia (52.26%), followed by Europe (21.8%), Latin America (10%), North America (9.1%), Africa (5.7%) and Oceania (0.87%). The difference in cancer incidence in these areas can be attributed to disparity in the socioeconomic status. HDI is a composite indicator that assesses human development in the countries. The index demonstrates a country’s status in three essential aspects of development, including life expectancy, education, and decent standards of living. Life expectancy is measured by the average period that a person is expected to live. Education is assessed based on years of potential education, and standards of living are measured based on per capita income or gross domestic product (GDP). In countries with high levels of HDI, the incidence and mortality of brain cancer are higher. This could be attributed to early screening, accurate healthcare services, early-stage diagnosis of disease, and a precise registry system in these countries [31].

The highest incidence of brain cancer has been observed in Australia, North America and Northern Europe in both sexes [18]. The highest mortality rates have been reported in China, the United States, India, Brazil and Russia [32]. The incidence and mortality rates of brain cancer has dropped in Western countries, but they are still on rise in some developing countries in Europe and Asia [33]. In European countries, the highest incidence rate was reported in England, Ireland and Northern Europe and the lowest in Eastern Europe [34].

Khodamoradi et al. (2017) reported that the highest incidence and standardized mortality rate of brain cancer belonged to Albania and there was a significant positive correlation between HDI (income level) and brain cancer incidence [15]. In the study of Davis et al. on patients with malignant brain tumor, 2 and 5-year survival rate were 36.2 and 27.6% respectively, which reflects a high mortality rate in low-income countries. This study manifested a significant positive correlation between HDI and brain cancer [35].

A 2016 study by Miranda et al. showed a significant positive association between HDI components and brain cancer (*P* < 0.001). Brain tumor incidence was lower in low-income individuals [36]. The findings of this study are consistent with results of our study. The above studies suggest that cancer is still a major global health issue in communities and more extensive studies are required to investigate the link between economic development and brain cancer.

Socioeconomic developments have been significantly associated with the incidence and attenuation of cancer. In low- and middle-income economies, the risk of cancers is on rise. Infections, as the leading cause of cancers, account for about 26% of cancers in middle- and low-income countries. Increased life expectancy, aging, lifestyle changes and exposure to biological hazards are risk factors for brain cancer [15]. Brain cancer is associated with industrialization in societies, and global health statistics suggest that most of cancer-induced deaths occur in developing countries.

Our results also exhibited that HDI was significantly correlated with the incidence (R = 0.690, *P* < 0.0001) and mortality of brain cancer (R = 0.629, *P* < 0.001) in 2018. The higher incidence of brain cancer in countries with higher HDIs could be ascribed to appropriate infrastructure, advanced diagnostic techniques, convenient access to primary healthcare, high quality of life, and early screening. Important risk factors for cancers in most countries are occupational and environmental hazards, ionizing radiation, and radioactive radiation. Early prevention strategies for brain cancer, such as diminished exposure to occupational hazards in the workplace and magnetic field exposure, can be effective in lowering the risk of cancers. However, considering the association between risk factors and outcomes, the results should be interpreted with caution, because besides the epidemiologic risk factors, the inherent limitations of ecological studies must also be taken into account. As the incidence and mortality of brain cancer are on rise in many countries, early preventive strategies, timely treatment, and patients’ follow-up, especially in less- developed countries, can help mitigate the disease burden and improve the health of people in these countries.

## Conclusion

According to the results of present study, the incidence and mortality rates for brain cancer are higher in countries with a higher HDI. The higher incidence rate in countries with a higher HDI can be explained in terms of environmental pollutants and occupational exposures to ionizing radiation and industrial radioactive sources in these countries. On the other hand, it could be argued that the higher rate of cancer in these countries may be due to more advanced facilities that can diagnose the disease. Therefore, further epidemiological studies about the factors related to the incidence and mortality of this disease can be effective in reducing its incidence and mortality rates.

### Strengths and limitations of the study

Since the cancer information registration system in all countries of the world is not collected with the same degree of accuracy, so the incidence data in each country may be affected by the information system of that country and the accuracy of the incidence and mortality rate in each country can be different.

## Data Availability

The datasets generated during the present study can be provided by the corresponding author upon reasonable request.

## Abbreviations

HDI: Human development index
NCDs: Non-communicable diseases
GNI: Gross national income
LEB: Life expectancy at birth
GDP: Gross domestic product
